# Scaling up community-based obesity prevention in Australia: Background and evaluation design of the Health Promoting Communities: Being Active Eating Well initiative

**DOI:** 10.1186/1471-2458-10-65

**Published:** 2010-02-12

**Authors:** Andrea M de Silva-Sanigorski, Kristy Bolton, Michelle Haby, Peter Kremer, Lisa Gibbs, Elizabeth Waters, Boyd Swinburn

**Affiliations:** 1 WHO Collaborating Centre for Obesity Prevention, Deakin University, Geelong, Australia 3217; 2 Victorian Department of Health, 50 Lonsdale St, Melbourne, Australia 3000; 3 School of Psychology, Deakin University, Geelong, Australia, 3217; 4 Melbourne School of Population Health, University of Melbourne, Parkville, Australia

## Abstract

**Background:**

There is only limited evidence available on how best to prevent childhood obesity and community-based interventions hold promise, as several successful interventions have now been published. The Victorian Government has recently funded six disadvantaged communities across Victoria, Australia for three years to promote healthy eating and physical activity for children, families, and adults in a community-based participatory manner. Five of these intervention communities are situated in Primary Care Partnerships and are the subject of this paper. The interventions will comprise a mixture of capacity-building, environmental, and whole-of-community approaches with targeted and population-level interventions. The specific intervention activities will be determined locally within each community through stakeholder and community consultation. Implementation of the interventions will occur through funded positions in primary care and local government. This paper describes the design of the evaluation of the five primary care partnership-based initiatives in the *'Go for your life' Health Promoting Communities: Being Active Eating Well *(HPC:BAEW) initiative.

**Methods/Design:**

A mixed method and multi-level evaluation of the HPC:BAEW initiative will capture process, impact and outcome data and involve both local and state-wide evaluators. There will be a combined analysis across the five community intervention projects with outcomes compared to a comparison group using a cross-sectional, quasi-experimental design. The evaluation will capture process, weight status, socio-demographic, obesity-related behavioral and environmental data in intervention and comparison areas. This will be achieved using document analysis, paper-based questionnaires, interviews and direct measures of weight, height and waist circumference from participants (children, adolescents and adults).

**Discussion:**

This study will add significant evidence on how to prevent obesity at a population level in disadvantaged and ethnically diverse communities. The outcomes will have direct influence on policy and practice and guide the development and implementation of future obesity prevention efforts in Australia and internationally.

**Trial registration:**

ACTRN12609000892213

## Background

Obesity is a growing public health issue and there is now widespread agreement that the complex etiology of obesity requires a multifaceted approach to prevention [1-4]. Community-based interventions provide an opportunity for community assets to be utilized with efficiency and direction [3]. There is now emerging evidence of the effectiveness of community-based and community-wide, multi-strategy approaches to obesity prevention [1,5-9].

A community-based, capacity-building approach aims to promote sustainable skill development, strengthen communities and increase the ability of individuals to effectively address and improve health outcomes [10,11]. Such an approach has the potential to influence the underlying social and economic determinants of health in a flexible, sustainable, equitable and safe manner [12]. To determine the sustainability of effective intervention activities, evaluation is required both in the intermediate and long term [13].

The aim of this paper is to describe the evaluation design of the five Primary Care Partnership (PCP)-based intervention sites in the *Health Promoting Communities: Being Active Eating Well *(HPC:BAEW) initiative to promote healthy eating and physical activity in disadvantaged communities across Victoria, Australia. The intervention will operate at a whole-of-community level within five intervention sites using a multi-setting, multi-strategy capacity-building approach. The evaluation objectives are to: 1) assess the extent to which the initiatives have been implemented as planned (process evaluation), and 2) evaluate the impacts and outcomes of the intervention when compared to a comparison sample.

## Methods/Design

### Intervention communities

Each intervention community has a specific primary target population, which ranges from children aged 0-12 years (including primary/elementary school students); to adolescents aged 12-18 years (secondary school students) and working adults. Secondary target populations include the wider community, older adults and specific disadvantaged groups (e.g. low income, ethnic minority, rural) within the community. The primary and secondary target groups for each intervention community are shown in Table 1, along with the characteristics of the communities.

**Table 1:** Intervention community characteristics and target groups.

Intervention Community	Community characteristics	Primary target group	Secondary target group
**1**	Rural and urban townships High percentage of socio-economic disadvantage	Primary school aged children	Families, carers, older adults and seniors
**2**	Urban Culturally and linguistically diverse Socio-economic disadvantage	Children 0-12 years	Families, carers, older adults and seniors
**3**	Rural townships High percentage of socio-economic disadvantage Significant proportion of young people	Secondary school aged children	Older adults
**4**	Urban Culturally and linguistically diverse High percentage of socio-economic disadvantage	Secondary school aged children	Young people newly arrived from overseas
**5**	Rural Culturally homogenous Ageing population group High percentage of socio-economic disadvantage	Working adults	Wider community

### Preparation for evaluation

The evaluation was not funded until after the funding and initiation of the five intervention projects. Therefore, prior to developing the evaluation plan, draft project action plans were reviewed in detail in conjunction with the project coordinators and affiliates for each of the projects and a matrix was developed to provide an overview of the implementation activities in each community. This is presented in Table 2.

**Table 2:** The intervention approaches for the objectives of the five Health Promoting Communities: Being Active Eating Well intervention communities*.

	Intervention approach				
Intervention objectives	Capacity building	Policy development & implementation	Community strengthening	Health skills & action competencies	Social marketing
Increase consumption of healthy food at home and/or in the community	2,4	4	2	2,4,5	2
Increase consumption of and access to fruit and vegetables	2,3,4	2,3,4	2,3	2,3,4	2,3,4
Decrease consumption of high fat, sugar, salt and energy dense foods	2,3	1,2,3	2,3	1,2,3	1,2,3
Increase water consumption	1,2,3,4	1,2,3,4		1,2,3,4,5	1,2,3,4
Decrease consumption of high sugar drinks	1,2,3,4	1,2,3,4		1,2,3,4	1,2,3,4
Increase the number of recreational opportunities for students/adults	3,4,5		2,3	3,5	5
Increase opportunities for formal and/or informal physical activity	2,3,4	2,3	2,3	2,3,4	2,3,4
Increase active transport		3		3,5	1,2
Increase awareness of healthy eating and physical activity guidelines	4,5			1,2,4	1,2,3,5
Increase initiation and duration of breastfeeding				2	
Improve healthy lifestyle using intergenerational and role modeling approaches	1		1	1,2	1,2
Foster positive body image	3			3	3
Community capacity building (leadership, partnerships, infrastructure etc)	1,2,3,4,5	2	1,5		

*The numbers relate to the specific intervention communities (1-5), see table 1 for more details.

**Figure 1:**
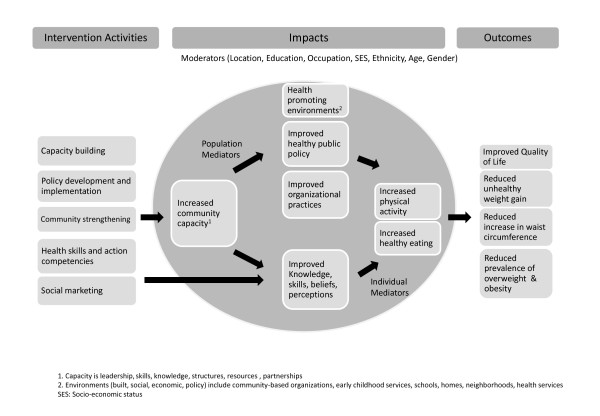
**Program logic model of the Health Promoting Communities: Being Active Eating Well initiative**.

A logic model was also developed on the basis of the action plans and frameworks being utilized to guide the intervention activities (see Figure 1). This logic model extended comparable models developed for other similar community-based projects, and provides a practical method for systematically collecting evaluation data for community projects [14].

### Evaluation design

The evaluation design will be quasi-experimental, repeat cross-sectional, with outcomes compared between intervention and comparison areas at two time points within the project duration (2008/9 and 2010). The socio-ecological model of health has been utilized to develop the intervention activities and evaluation design. This framework focuses attention on five key determinants of health behavior: individual factors, interpersonal and primary groups; institutional and organizational factors, community factors and public policy [15]. The model assumes that appropriate changes in the social environment will produce changes in individuals and the support of individuals in the population is essential for implementing environmental changes [16]. Consistent with this framework, impact and outcome data will be collected at the community, setting and individual levels. Table 3 provides an overview of the evaluation data to be collected.

**Table 3:** Overview of the multi-level and mixed methods evaluation data to be collected

Level	Details	Tools
**Community**	Networks and partnerships Opportunities for physical activity Access to nutritious food Policy development and implementation Capacity building	Partnership/community assessment Document analysis Key informant interviews (post intervention)
**Workplace**	Environment Access to healthy food Opportunities for physical activity Policy development	Environment assessment Document analyses
**School**	Socio-cultural environment Policy development and implementation Capacity building Access to nutritious food Opportunities for physical activity	School environment assessment
**Individual**	Food and activity related behaviors Weight status Quality of life	Health behavior questionnaire Anthropometric Assessment Quality of Life Questionnaire

### Comparison groups

Appropriate comparison groups for each target (age) group will be selected from across the state. Comparison schools will be randomly drawn from Victorian schools matched on demographics including school type (government/non-government), school size, level of disadvantage (using the Socio-Economic Index for Areas [SEIFA] index of advantage/disadvantage Victorian decile from the 2006 Census [17]) and location (Local Government Area and region). Comparison workplaces will be matched on the demographic characteristics of the intervention workplaces, including the workplace size, type and location.

### Sample size

The sample size calculations were primarily designed to account for intervention outcomes and detect a meaningful level of change compared to the comparison group. As such, calculations were based on a difference in behavior prevalence of > 15% and zBMI > 0.15 units between intervention and comparison groups, while also accounting for the design effects of clustering by schools and workplaces. Detecting differences within this design will require a sample of approximately 5,200 students (approximately 2,400 primary students and 2,800 secondary students). For the adult sample, 245 people in the intervention and a matched comparison sample will have the power to detect a 13 percentage point difference in behavioral outcomes (power of 80%) and difference of 1.7 BMI units between intervention and comparison samples with 80% power at a significance level of 0.05.

### Process evaluation

The primary methods to assess the extent to which the intervention activities have been implemented and determine program reach (process evaluation) will include: document analysis (e.g. meeting minutes, action plan versions, reports, school/workplace policies, food service menus, and curriculum); key informant interviews; participant feedback; focus groups; case studies of participants/community organizations and surveys. At a local level, project managers and workers will also capture the approaches to intervention development and promotion, participant recruitment, level of (and explanation for) participation and non-participation, demographics of participants (e.g. gender, socio-economic position, age group) and any publicity or press for the local project. Together this data will comprise the process evaluation.

### Impact and outcome evaluation

Differences in individual and community-level impacts and outcomes will be measured using a cross-sectional design with differences between intervention and comparison groups assessed at the cluster (school, workplace, community) level as appropriate. Trained research staff (supported by project staff at baseline) will collect the anthropometric, behavioral, quality of life and environmental data from each school/workplace pre- (2008/9) and post- (2010) intervention activities. Data on socio-demographics will also be collected in two ways: direct data collection from the participant (age, gender, country of origin, residential address, school address) and from the community (region) using the 2006 ABS Census data (e.g. mean income, education, occupation, indigenous status, family structure, country of origin, English-speaking). This community level data will be used in hierarchical linear modeling to analyze the outcomes of the interventions. At the local level, additional intervention impacts will be captured through qualitative methods (narrative evaluation, case studies, photo diaries etc).

### Anthropometry

Weight, height and waist circumference will be measured by trained researches in accordance with standard methods for the collection of anthropometric data [18]. All measurements will be taken in light clothing (one layer), without shoes, with all jewellry removed and pockets emptied. Cultural and body image sensitivities will be accommodated using previously published strategies [19]. Weight will be measured to the nearest 0.05 kg using electronic scales (A&D Personal Precision Scale UC-321). Height will be measured using a portable stadiometer to the nearest 0.1 cm (PE87 portable stadiometer) and participant hair styles which may interfere with the measurement process will be removed or adjusted. Participants will be instructed to stand with their weight distributed evenly on both feet, with their heels together and arms hanging freely by their sides. Four contact points between the participant's body and the measuring apparatus will be required (head, upper back, buttocks and heels) and the head aligned in the Frankfurt plane prior to the measurement being taken. Waist circumference will be measured to the nearest 0.1 cm at the end of a normal expiration on the horizontal line of the umbilicus using a standard flexible seamstress measuring tape for students and a constant tension tape figure finder for adults. Two measurements will be recorded for each parameter, and where there is a disagreement between two measurements (>0.1 kg for weight, >0.5 cm for height and >0.3 cm for waist circumference), a third will be taken. The mean of all measures will be used for analysis, and Body Mass Index (BMI) (weight in kg/height in m^2^) will be calculated to classify child and adult weight status using the International Obesity Task Force age-specific BMI cut-off points [20]. The scales and stadiometer will be re-calibrated every 1000^th ^student measured and the flexible measuring tape verified against a designated 1 m metal ruler on a weekly basis to confirm the tape has not stretched.

### Survey methodology

Surveys capturing information regarding nutrition and physical activity behaviors will be piloted and subsequently administered to all students in grade four and higher, and to all adults. Surveys will be based on those used in our previous and similar studies [12] and http://www.goforyourlife.vic.gov.au/hav/articles.nsf/practitioners/Be_Active_Eat_Well_Final_Reports?Open) and aligned with state-wide surveillance measures where possible.

### Nutrition and physical activity behaviors

The behavioral surveys will contain 20 to 30 questions primarily focusing on the types and amounts of foods eaten on the previous day and the type, frequency and duration of sedentary behaviors and physical activity. Age-appropriate surveys will be used for younger and older children and adults.

### Quality of life

Secondary school students and adults will also complete the Assessment of Quality of Life (AQoL) mark 2 [21,22], a 20 item quality of life assessment tool comprising six dimensions: physical ability, social and family relationships, mental health, coping, pain and physical senses (vision, hearing and communication). Scores from the six dimensions will be combined to calculate an overall quality of life rating. The AQoL, is a utility-based instrument originally developed for Australian adults, which has now been recalibrated for use with adolescents (M. Moodie, personal communication, manuscript under review).

### School environment

An assessment of the school environment will be conducted by a trained researcher with one to three school staff to complete a questionnaire designed to capture seven key elements of the school: demographics, internal and external food services, food/nutrition and physical activity policy(ies); nutrition environment; and the physical activity environment. The questionnaire is based on similar instruments that have been developed and used for environment audits [23] and a consensus answer will be recorded for each question.

### Measures of deprivation and ethnicity

The postcode of each participant will be used to determine the SEIFA This is an area-level indicator of socio-economic status, with a low score on the SEIFA indicating an area of social disadvantage and a higher score an area less disadvantaged [17]. Information related to ethnicity and length of time since migration to Australia will also be collected.

### Data entry, handling and statistical analysis

The child behavioral surveys will be scanned directly into an electronic database. The demographic, other survey (adult behavioral and AQoL) and anthropometric data will be double-entered into Stata (Version 10.1, StataCorp, College Station, Texas, USA). All statistical analysis on individual and school environment data will be conducted using Stata and quantitative analysis of the cross-sectional impact and outcome data will utilize regression analysis, hierarchical linear modeling, descriptive statistics and design corrected chi^2 ^analysis, as appropriate. The SVY commands in stata will be used for all analysis to account for clustering and design effects and the primary analysis will be between the intervention and comparison groups post-intervention. Demographic data will be used to adjust for confounding (e.g. gender; socio-economic status), and additional data on smoking status and alcohol intake will be collected from adults. Qualitative data will be analyzed using content and inductive thematic methods, as appropriate. Data triangulation and mixed methods analysis will also be used where possible.

### Consent and ethics

All adults participating in the evaluation will provide informed written consent. For younger participants (aged <18 years), parents will provide informed written consent and the participant will provide additional verbal consent prior to data collection. Various strategies will be used to maximize the response rate of participants. The project has been approved by the Deakin University Human Research Ethics Committee (HREC, EC98-2008), the Department of Health HREC, relevant hospital HRECs, the, Department of Education and Early Childhood Development (DEECD), the Office for Children Research Coordinating Committee, and the Catholic Education Offices (Melbourne and regional) as appropriate. All researchers involved in data collection will have a Victorian Police Check and/or a "Working With Children" (WWC) check.

## Discussion

Currently, there is only limited evidence available about effective strategies to prevent obesity at a population level. The *Health Promoting Communities: Being Active Eating Well* initiative is an attempt to scale up intervention activities across multiple communities in a coordinated manner using a capacity-building and environmental focused approach. The evaluation of the initiative is challenging due to the late contracting of the evaluators and the necessity to co-ordinate the evaluation across multiple communities with varying target groups and intervention activities. Despite these challenges, the knowledge generated will add significantly to the evidence base and can inform future large and small scale obesity-related public health interventions internationally.

## Competing interests

The authors declare that they have no competing interests.

## Authors' contributions

AdS and KB drafted the initial manuscript, all authors contributed to the methods and design of the study, and all authors had critical input into the production of the final manuscript. All authors have read and approved the final manuscript.

## Pre-publication history

The pre-publication history for this paper can be accessed here:

http://www.biomedcentral.com/1471-2458/10/65/prepub
